# A Pilot Study to Investigate the Role of Thymidylate Synthase as a Marker of Prognosis for Neoadjuvant Chemotherapy in Gastric and Gastro-Oesophageal Junction Adenocarcinoma

**DOI:** 10.1155/2013/502153

**Published:** 2013-03-03

**Authors:** A. Mirza, M. Brown, C. McNulty, J. Valentine, A. Annesley, S. Galloway, I. Welch, C. M. West, S. Pritchard

**Affiliations:** ^1^Departments of Gastrointestinal Surgery and Histopathology, University Hospital of South Manchester, Manchester M23 9LT, UK; ^2^Translational Radiobiology Group, School of Cancer and Enabling Sciences, The University of Manchester, Manchester Academic Health Science Centre, Christie Hospital NHS Trust, Manchester M20 4BX, UK; ^3^Departments of Surgery and Histopathology, The University Hospital of South Manchester, Southmoor Road, Manchester M23 9LT, UK

## Abstract

*Aims and Background*. Patients in the United Kingdom with operable gastric and gastro-oesophageal junction (GOJ) tumours receive neoadjuvant chemotherapy. Our aim was to study the expression of thymidylate synthase (TS) enzyme in pre-treatment diagnostic biopsy specimens and investigate its clinical usefulness. *Methods*. A single-centre study was carried out in 45 patients with gastric and GOJ adenocarcinoma treated with neo-adjuvant chemotherapy according to the MAGIC protocol. TS expression was determined using immunohistochemistry. >10% tumour nuclei expression of TS was used as cut-off for positivity. *Results*. Forty-one (91%) of the 45 tumours expressed TS. There was no association between TS expression and lymph node status (*P* = 0.80), histological response (*P* = 0.30), and recurrence (*P* = 0.55). On univariate analysis, only N-stage (*P* = 0.02) and vascular invasion (*P* = 0.04) were associated with a poor prognosis. Patients with negative tumour TS expression had better outcome than those with positive expression. The overall 5-year survival rate was 100% in the TS negative versus 56% in TS positive group, but the difference was not statistically significant (*P* = 0.17). *Conclusion*. TS expression should be studied in a larger series of gastro-oesophageal cancers as a potential prognostic marker of prognosis to neo-adjuvant chemotherapy.

## 1. Introduction 

Gastric and oesophageal cancers are associated with poor five-year survival rates [1–3]. Potentially curative treatment involves both pre- and postoperative chemotherapy often combined with radiotherapy as most studies support the use of neoadjuvant and adjuvant chemotherapy to improve prognosis [4]. Most patients show either no or partial response, despite receiving the full course of neoadjuvant chemotherapy (UK) or up to 50% response in combination of neoadjuvant chemoradiotherapy (USA, Europe). This ineffective treatment delays potentially curative surgical resection in these patients and is often associated with disease progression. Neoadjuvant chemotherapy is also associated with toxicity, that is, anaemia, neutropenia, thrombocytopenia, and nausea [5]. Solid cancers differ in their genetic makeup and even the same histologic subtypes of cancer show a wide spectrum of variation in results achieved following administration of chemotherapeutic agents [6]. 

The 5-FU-based chemotherapy is the corner stone of cancer treatment and is used in the first-line management of several solid tumours including gastro-oesophageal cancers [7, 8]. The 5-FU is a fluorinated nucleotide base analogue and an antimetabolite that inhibits both DNA and RNA synthesis [9]. It targets tumour cells because they generally have a higher metabolism and rate of proliferation than cells in normal tissue [10]. The 5-FU undergoes multiple intracellular changes before inhibiting proliferation. TS is a rate-limiting enzyme in the phosphorylation of 5-dUMP to 5-dTMP. The 5-dTMP under normal conditions is converted to 5-dTTP which is incorporated into DNA during its routine synthesis. In the absence of TS DNA synthesis is inhibited and this results in cell death [6]. Increased expression of TS in the tumour cells enables them to overcome 5-FU-induced nuclear cytotoxicity and promotes DNA synthesis and repair of tumour cells. 

In oesophageal cancer, increased TS expression has been recognised to be associated with poor differentiation [11], nonresponse to chemotherapy [12], and tumour recurrence [13]. In gastric cancer TS expression has been studied as a predictive marker of response to chemotherapy [14–16] and poor differentiation [17]. Patients in the United Kingdom with operable gastric and gastro-oesophageal junction (GOJ) tumours receive neoadjuvant chemotherapy prior to surgical resection according to the United Kingdom Medical Research Council Adjuvant Gastric Infusional Chemotherapy protocol [7]. To date, no study has assessed the ability of TS expression to predict benefit from neoadjuvant chemotherapy given according to the MAGIC protocol.

The aim of this study was to investigate TS expression as a prognostic and predictive biomarker in pretreatment diagnostic biopsy specimens of gastric and gastro-oesophageal junction adenocarcinoma in patients who received neoadjuvant chemotherapy according to the MAGIC protocol. This study was designed as a pilot project on a consecutive cohort of patient from a single centre. In this pilot study if immunohistochemical staining was successfully performed and an association of TS expression with clinicopathological features was identified, a larger cohort of patients from several centres will be included in the second phase to determine its clinical application.

## 2. Materials and Methods 

### 2.1. Patients and Tissue Specimens

Ethical approval for the study was obtained from the North West Research Ethics Committee, Greater Manchester, UK (09/H1014/63). The study was carried out in 45 consecutive patients with locally advanced gastric and GOJ adenocarcinoma treated with neoadjuvant chemotherapy followed by surgical resection at The University Hospital of South Manchester (UHSM) between 2002 and 2006. Inclusion criteria were as follows: adenocarcinoma, locally advanced disease, fit for surgical resection, and at least one course of neoadjuvant chemotherapy. All patients underwent a staging protocol of CT scanning, endoscopic ultrasonography, and staging laparoscopy. The patients received neoadjuvant chemotherapy according to the MAGIC protocol [7]. The chemotherapy was administered for three cycles pre- and postoperatively. A cycle consisted of epirubicin (50 mg/m^2^) by intravenous bolus and cisplatin (60 mg/m^2^) intravenously with hydration on day one and 5-FU (200 mg/m^2^) daily for 21 days by continuous intravenous infusion. All patients underwent a restaging CT scan of the abdomen and chest postneo-adjuvant chemotherapy to assess for disease spread and potential disease downstaging. Patients with gastric cancer underwent a partial or total gastrectomy depending on the site of the tumour along with D2 lymphadenectomy. Patients with a GOJ tumour underwent either Ivor-Lewis oesophagectomy (type I Siewert) or extended total gastrectomy (type II or III Siewert). The surgical excision was performed by two surgeons. Following surgery patients were treated with three further cycles of chemotherapy. The demographic details of patients which included gender, age, and status at last followup were collected. 

### 2.2. Assessment of Histopathological Response

For the assessment of histopathological response following neoadjuvant chemotherapy paraffin blocks and haematoxylin and eosin (H&E) stained sections stored from resection samples were obtained. If H&E sections were deemed to be of poor quality, fresh sections were cut. The histology slides were reviewed independently by two histopathologists. The degree of histological response to neoadjuvant chemotherapy was assessed employing Becker's criteria [18]. Grade 1A: no residual tumour/tumour bed; Grade IB: <10% tumour cells; Grade 2: 10–50% residual tumour/tumour bed; Grade 3: >50% no signs of neoplastic regression. Grades 1A and 1B are classed as responders while Grades 2 and 3 are classed as non-responders.

### 2.3. Immunohistochemical Staining and Scoring

The formalin-fixed, paraffin wax embedded (FFPE) diagnostic blocks were obtained from seven hospitals which referred patients for surgical management at UHSM. These blocks contained the first diagnostic specimens obtained prior to initiation of neoadjuvant chemotherapy. Sections 4 *μ*m thick were cut, placed on slides, heated to 60°C to ensure adhesion, dewaxed in xylene for 15 min, and dehydrated and dewaxed in 99% alcohol (industrial methylated spirit) for 9 min. Peroxidase activity was then blocked by exposure to 3% hydrogen peroxide solution (450 ml of 99% industrial methylated spirit and 50 ml of concentrated 30% hydrogen peroxide) for 10 min. After washing in water for 5 min, sections were heated in a pressure cooker in 0.01 M citrate buffer (3 litres of deionised water to 30 ml of citrate buffer at pH 5.5) for 2 min followed by washing with tris-buffered saline (TBS) pH 7.6 with tween (diluted 1 in 10 with deionized water). Positive control (primary colorectal tumour) and the test tissue sections were then incubated with a monoclonal mouse antihuman TS antibody (TS 106, Dako, Glostrup, Denmark; diluted 1 : 100) for 30 min at room temperature. The negative control solution (Dako antibody and horse serum) was applied to the negative control tissue section for 30 min. Sections were then washed with TBS buffer, treated with diaminobenzidine for 5 min, washed with deionised water, counterstained with Mayer's haematoxylin, dehydrated, and coverslipped.

**Figure 1:**
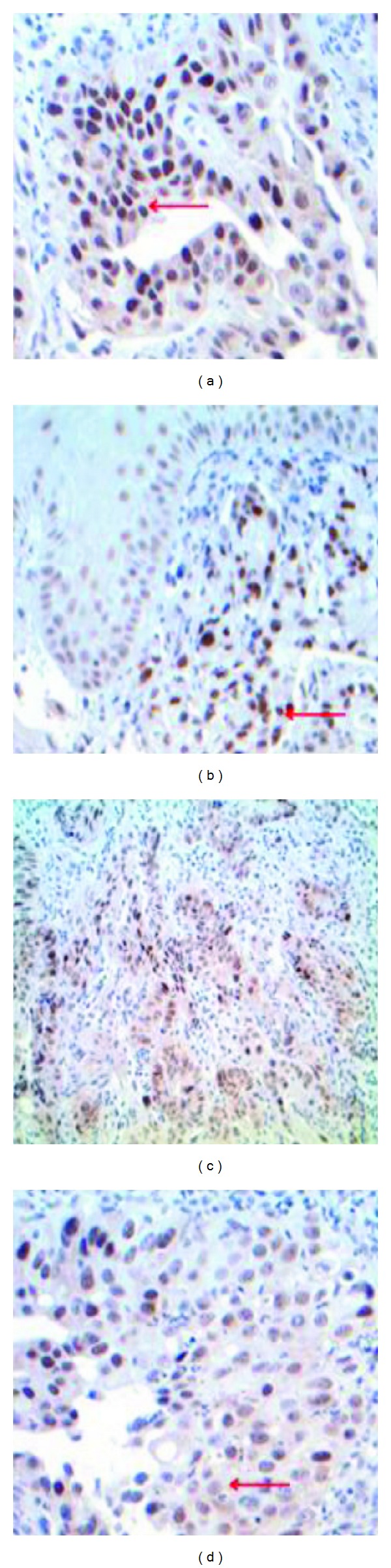
Immunohistochemical staining for TS expression. (a) Expression in the nucleus of tumour cells (red arrow ×200). (b) Expression in tumour and normal gastric cells ×40. (c) Moderate expression in tumour cells ×40. (d) Weak/moderate staining in the cytoplasm of tumour cells (red arrow ×200).

Different methods have been described for scoring TS expression with no consensus available. Various cut-off levels have been used to determine TS expression in tumour cells: median [19]; 10% [20]; 30% [21]; or 50% [14] positive cells. As the same antibody was used, we used the method described by Langer et al. and staining of >10% of tumour cells was considered positive [20].  Figure 1 illustrates the pattern of staining for TS expression. TS was expressed in both normal gastric and tumour cells. Only tumour cell staining was scored. At the cellular level TS expression was observed both in the nucleus and the cytoplasm. In the literature, variations are observed in scoring for TS: nuclear [20], cytoplasmic, [19] and both nuclear/cytoplasmic [11]. In our study only the nuclear staining was considered. Tumour cells were also scored for intensity of staining graded as weak, moderate, or strong. Scoring was carried out independently by two observers and a consensus score was agreed. There was an acceptable degree of agreement between the two observers (kappa score = 0.61) before a consensus was agreed.

### 2.4. Statistics

SPSS version 16 (SPSS, Inc, Chicago, IL, USA) was used for statistical analysis. Pearson chi-square or Fisher's exact tests were used to study associations between immunohistochemical staining and multiple histopathological factors. Survival curves were plotted using the method of Kaplan-Meier and log-rank tests were employed to determine the degree of significance. A *P* value of <0.05 was considered statistically significant. Both univariate and multivariate analyses were performed using the Cox-proportional hazards model. Overall survival was calculated from the date of surgery to the date of last followup or death. The progression-free survival was calculated from the start of neoadjuvant chemotherapy to the first event (local recurrence, distant recurrence, and death from any cause).

## 3. Results

**Figure 2:**
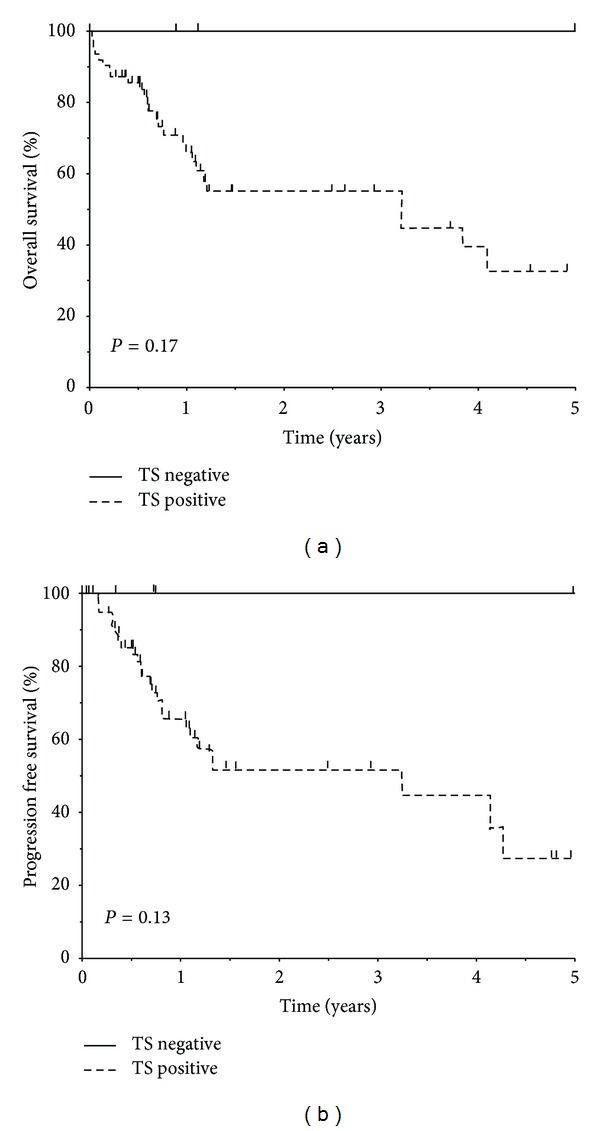
Progression-free and overall survival stratified by tumour TS expression. Four patients with low TS expression had a better outcome than patients with high tumour TS expression.

Data were collected for 45 patients suffering from gastric and GOJ cancer who received neoadjuvant chemotherapy prior to surgical resection.  Table 1 summarises the patient characteristics. The median patient age was 64 (range 47–77) years. The average number of preoperative cycles received was 2 (range 1–3). The mean time from diagnosis to surgery was 5 months (95% confidence interval (CI), 2 to 7). Tumour recurrence was documented in 16 (36%) patients. The mean time for tumour recurrence was 19 months (95% CI, 13 to 25 months). The mean survival time from date of surgery was 20 months (95% CI, 14 to 26 months). The level of TS expression ranged from 0 to 100%. The median TS expression score was 65% (range 0 to 100%). Using the criterion of >10% positive tumour cells determining positivity, 41 (91%) of the patients had TS-positive diagnostic biopsies. The TS intensity scores were weak in 7 (17%) and moderate/strong stain in 34 (83%). All TS-positive cases showed weak/moderate staining in the cytoplasm and medium/strong staining in the nucleus of the tumour cells. There was uptake of TS stain by the normal cells but the intensity of stain uptake was significantly higher in the tumour cells (*P* = 0.02). A complete or near-complete histological response was seen in only 4 (9%) patients who had total or near-total absence of viable tumour cells in samples from resection specimens. There were no associations between TS expression and clincopathological features (Table 2).  Table 3 summarises the results of univariate analyses. On univariate analysis both nodal stage (*P* = 0.02) and vascular invasion (*P* = 0.04) were identified as significant prognostic factors while on multivariate analysis only nodal stage emerged as an independent prognostic factor (HR = 2.56, 1.53–4.32, *P* = 0.02).  Figure 2 shows five year progression-free and overall survival for patients stratified by TS expression. The two year progression-free survival was 58% but was not significantly different between the two groups. The overall 5-year survival rate was 100% in the TS negative compared with 56% in the TS positive group, but the difference was not statistically significant (*P* = 0.17). The analyses were repeated using the median level of TS expression as a cut-off value but no statistically significant results were obtained. 

## 4. Discussion 

Our study found 41 (91%) biopsy specimens showed positive tumour nuclear staining for TS. It is difficult to compare the level of expression seen with other values reported in the literature because of the variety of approaches used to determine positivity. TS expression was reported in 56% (64/114, semiquantitative) of oesophageal adenocarcinoma [13]; 66% (82/124, ≥20% cytoplasmic immunostaining taken as positive) of stomach cancers [16]; and 61% (19/31, same method as used here) of oesophageal adenocarcinoma [20]. In colorectal cancers, positive TS expression was seen in 72% (Grades 2, 3 versus 0, 1) [22] and 70% (semiquantitative, 0–2/3-4) [23] of patients. Although the 91% positivity found in this study is high it broadly agrees with the high level of expression reported by others. This variation in positivity is probably due to differences in the tumour type studied, type of antibody used, immunohistochemical methodology, and scoring techniques for expression (cut-off values, grading criteria, and semiquantitative method).

Our study found no association between the level of TS expression and histological response to neoadjuvant chemotherapy. There was global expression of TS observed both in the cytoplasm and the nuclei of the tumour cells. This may also represent the increased TS activity in the gastro-oesophageal adenocarcinoma. Due to this high expression in 91% of the biopsy specimens no significant association with other clinicopathological features was observed, but the number of negative staining cases was too small to draw any firm conclusions. There was a strong trend for TS positive tumours having a poor prognosis, however, which is consistent with the published literature. High TS expression was associated with a poor response to neoadjuvant chemotherapy in patients with oesophageal adenocarcinoma [20]. A pancreatic cancer study showed patients with high tumour TS expression benefited from adjuvant chemotherapy and low TS level was associated with improved overall survival [24]. In colorectal cancer, multiple studies showed increased tumour expression of TS was associated with a poor prognosis. A meta-analyses of 20 studies (*n *= 3497) in the advanced and adjuvant settings in colorectal cancer concluded that high TS levels was associated with a poor overall survival [25]. The meta-analysis described quantitative and semiquantitative methods for the assessment of TS expression but no single universally acceptable method for staining and scoring of TS expression was described. In patients who have undergone only primary surgical resection for colorectal carcinoma without neoadjuvant or adjuvant chemotherapy increased TS expression was identified to be an independent prognostic factor for recurrence, metastases, poor overall and cancer-specific survival [26]. A large Scandinavian study (*n* = 862) of colorectal cancer patients undergoing potentially curative surgery showed a survival benefit when prescribing adjuvant 5-FU to patients with high tumour TS expression. There was no documented survival benefit rather an increase in co-morbidity when 5-FU was prescribed to patients with low tumour TS expression [22]. A meta-analysis of 24 published studies in colorectal cancer investigating TS expression identified low expression to be associated with increased 5-FU chemosensitivity [27]. The study grouped multiple studies employing RT-PCR and immunohistochemistry and also multiple TS expression scoring methods (median cutoff and semiquantitative). 

Other methods are being explored to assess TS in tumours. TS mRNA expression was identified as an independent prognostic marker in oesophageal adenocarcinoma [28] and a predictor of response to chemotherapy in inoperable gastric cancer [29]. mRNA analysis showed TS expression in oesophageal adenocarcinoma reduced postneo-adjuvant chemotherapy [12]. There is also interest in looking at polymorphisms in the TS gene. The human TS gene is located on chromosome 18p11.32 and is composed of seven exons [30]. There are tandem repeats of 28 bp in the TS gene which are located upstream of the transcriptional promoter. They contain tandem repeat sequences of 2R, 3R, and so forth. Multiple transcription regulatory proteins combine with the tandem repeat sequence to affect TS gene expression. The most common TS gene polymorphism are 2R/2R homozygote; 2R/3R heterozygote; and 3R/3R homozygote [15]. In recent years TS gene polymorphisms have gained intense focus as the key factor determining TS activity. In the literature, there are conflicting reports about the effect of TS polymorphisms. A study in gastric cancer patients showed 2R/2R, 2R/3R, and 3R/3R genotypes were associated with a good outcome [31]. A recent prospective rectal cancer adenocarcinoma study treated patients with neoadjuvant chemotherapy based on TS genotype. Patients with 2R/2R, 2R/3R, and 2R/4R genotypes were classed as low risk and treated with a standard protocol (5-FU + radiotherapy). Those with 3R/3R and 3R/4R genotypes were regarded as high risk and treated with additional chemotherapy. This aggressive treatment strategy based on genotype subgrouping resulted in downstaging and an improved rate of negative resection margins [32]. Another study on colorectal cancer, however, concluded TS genotype had no significant impact on outcome following 5-FU-based chemotherapy [33]. A study on metastatic gastric and colorectal cancer identified the 2R/2R TS genotype as being more sensitive to 5-FU activity [15]. The 2R/2R genotype was also identified as a risk factor for the development of gastric cancer [34]. 

## 5. Conclusion

In order to progress this work further, there is a need to develop internationally standardised methods for TS immunohistochemistry and mRNA analysis and a consensus on the best approach for determining tumour positivity. Given the marked trend observed in the survival data, a larger prospective study is warranted. Also it would be interesting to compare pre- and posttreatment TS expression in the tumour specimen to identify the response at the cellular level. 

## Figures and Tables

**Table 1:** Patient characteristics.

Characteristics	No. (%) of patients
Gender	
Male	33 (73%)
Female	12 (27%)
Tumour differentiation	
Well/moderate	29 (64%)
Poor	16 (36%)
Tumour site	
Gastric	17 (38%)
GOJ	28 (62%)
T stage	
0/1	22 (49%)
2/3/4	23 (51%)
N stage	
Node negative	14 (31%)
Node positive	31 (69%)
Vascular invasion	
No	38 (84%)
Yes	8 (16%)
Lymphatic invasion	
No	37 (82%)
Yes	8 (18%)
CRM	
Negative	35 (78%)
Positive	10 (22%)
Tumour recurrence	
Yes	29 (64%)
No	16 (36%)
Histological response	
Yes	4 (9%)
No	41 (91%)

CRM: circumferential resection margin.

**Table 2:** Clinical and histological factor associations by Fisher's Exact test.

Characteristics	TS positive	TS negative	Fisher's exact test
Gender			
Male	30	3	0.71
Female	11	1	
Tumour site			
Gastric	15	2	0.49
GOJ	26	2	
Differentiation			
Well/moderate	19	1	0.62
Poor	22	3	
T stage			
T0/T1	20	2	0.68
T2/3/4	21	2	
N stage			
Negative	11	3	0.08
Positive	30	1	
Vascular invasion			
Yes	6	1	0.51
No	35	3	
Lymphatic invasion			
Yes	7	1	0.56
No	34	3	
CRM			
Negative	31	4	0.56
Positive	10	0	
Histological response			
Yes	3	1	0.32
No	38	3	
Recurrence			
No	26	3	0.55
Yes	15	1	

*Fisher's exact test.

GOJ: gastro-oesophageal junction; CRM: circumferential resection margin.

**Table 3:** Univariate analysis of clinicopathological factors.

Characteristic		Overall survival		Cancer-specific survival	
		HR (95% CI)	*P*	HR (95% CI)	*P*
Gender	Male versus female	1.13 (0.37–3.47)	0.82	0.80 (0.21–3.04)	0.75
T stage	0/1/2 versus 3/4	0.95 (0.37–2.46)	0.91	1.08 (0.31–3.82)	0.90
N stage	Positive versus negative	2.36 (1.52–3.46)	0.02	3.95 (1.04–6.82)	0.04
Lymphatic invasion	Present versus absent	1.18 (0.38–3.66)	0.77	1.03 (0.27–3.96)	0.96
Vascular invasion	Present versus absent	2.91 (1.02–4.03)	0.04	1.55 (0.33–7.31)	0.35
CRM	Positive versus negative	0.76 (0.42–3.32)	0.76	1.71 (0.55–5.26)	0.14
Differentiation	Well/moderate versus poor	1.08 (0.42–2.82)	0.86	0.85 (0.28–2.56)	0.64
TS	Negative versus positive	1.32 (0.36–2.56)	0.81	1.27 (0.29–1.92)	0.76
